# Animal sentience

**DOI:** 10.1111/phc3.12822

**Published:** 2022-03-17

**Authors:** Heather Browning, Jonathan Birch

**Affiliations:** ^1^ Centre for Philosophy of Natural and Social Science London School of Economics and Political Science London UK

## Abstract

‘Sentience’ sometimes refers to the capacity for any type of subjective experience, and sometimes to the capacity to have subjective experiences with a positive or negative valence, such as pain or pleasure. We review recent controversies regarding sentience in fish and invertebrates and consider the deep methodological challenge posed by these cases. We then present two ways of responding to the challenge. In a policy‐making context, precautionary thinking can help us treat animals appropriately despite continuing uncertainty about their sentience. In a scientific context, we can draw inspiration from the science of human consciousness to disentangle conscious and unconscious perception (especially vision) in animals. Developing better ways to disentangle conscious and unconscious affect is a key priority for future research.

## 1 SENTIENCE AND WHY IT MATTERS

Sentience (from the Latin *sentire*, to feel) is an important concept in animal ethics, bioethics, and the science and policy of animal welfare. There are broader and narrower senses of the term. In a broad sense, sentience can refer to the capacity for *any type of subjective experience*: any capacity for what philosophers tend to call ‘phenomenal consciousness’ (Block, 1995; Nagel, 1974). An animal is sentient in this sense if, at least under the right conditions (e.g. when it is fully awake), there is ‘something it's like’ to be that animal.^1^

In a narrower sense, sentience can refer to the capacity to *have subjective experiences with positive or negative valence* ‐ experiences that feel bad or feel good ‐ such as pain, pleasure, anxiety, distress, boredom, hunger, thirst, pleasure, warmth, joy, comfort and excitement (e.g. DeGrazia, 1996; Duncan, 2006; Jones, 2013). In our own case, many of these experiences involve a mix of sensory, affective, and cognitive components (e.g., pain involves a sensation of injury at a specific location *and* an accompanying negative affect; Auvray et al., 2010), but it is the affective component of these experiences that makes them feel bad or feel good (Shriver, 2018). Accordingly, sentience in this narrower sense is sometimes also known as ‘affective sentience’ (Powell & Mikhalevich, 2021) and is very close to one important sense of the ordinary word ‘feeling’ (Harnad, 2016).^2^

The capacity to have valenced experiences has a special significance for ethics. It is often said to be a ground, or even *the* ground, for moral status. This idea goes back to Bentham's iconic dictum: ‘the question is not *Can they reason?* or *Can they talk?* but, *Can they suffer?*’ (Bentham, 1789). Singer (1979) defines sentience as ‘the capacity to suffer or experience enjoyment or happiness’ and clearly takes this capacity to be both necessary and sufficient for the possession of morally significant interests. This remains a popular view in animal ethics (DeGrazia, 1996; Varner, 2012). Empirical work also suggests many non‐philosophers also see the capacity for subjective experience as one factor (though not the only factor) justifying attributions of moral standing (Goodwin, 2015; Jack & Robbin, 2012; Sytsma & Machery 2012).

In recent decades, this concept of sentience has also come to carry great significance for animal welfare law in the UK, European Union (EU), Canada, Australia and New Zealand. The EU's Lisbon Treaty states that member states ‘shall, since animals are sentient beings, pay full regard to the welfare requirements of animals’ (Article 13, Title 2). The UK's Animal Welfare Act 2006 defines ‘animal’ as ‘a vertebrate other than man’, but adds that invertebrates could be brought within the scope of the Act ‘if the appropriate national authority is satisfied, on the basis of scientific evidence, that animals of the kind concerned are capable of experiencing pain or suffering’ (s1(4)). Quebec's Animal Welfare and Safety Act begins by noting that animals ‘are sentient beings that have biological needs’ (B‐3.1, p.1). New Zealand's Animal Welfare Act 1999 contains an opening clause to ‘recognise that animals are sentient’ (a(i)). In 2019, the Australian Capital Territory passed amendments to its Animal Welfare Act 1992 to formally recognise that ‘animals are sentient beings that are able to subjectively feel and perceive the world around them’ (s4A(1a)), which has influenced other states to discuss implementing similar changes. This legislative role has imbued debates about which animals are sentient (and which are not) with a sense of practical urgency ‐ and it is generally the capacity to have valenced experiences that is at issue in these debates.

The concept of sentience (usually in the narrower sense) also features in animal welfare science. Scientists in this area often take it for granted that, in studying welfare, the goal is to access (albeit indirectly) experienced affective states (e.g. Browning, 2020; Duncan, 2002; Kirkwood, 2006; Mellor et al., 2020). But not all animal welfare scientists are in agreement on this point. Notably, Dawkins (2012, 2017, 2021) has argued for a conception of welfare in which sentience plays no role. In Dawkins' view, animal welfare scientists have tended to grossly underestimate the difficulties of studying subjective experience in animals: having found evidence of an *affective state*, they immediately jump to the conclusion that the state is *consciously experienced*, without making any serious case for this. This is an illicit inference, given the evidence from humans that some affect is unconscious (Berridge & Winkielman, 2003; Paul et al., 2020). This issue in fact lies at the heart of current controversies regarding sentience, to which we will now turn.

## 2 CURRENT CONTROVERSIES

So, which animals are sentient? In 2012, the Cambridge Declaration on Consciousness (Low et al., 2012) crystallised a scientific consensus that humans are not the only sentient beings. It added that ‘non‐human animals, including all mammals and birds, and many other creatures, including octopuses’ possess neurological substrates complex enough to support conscious experiences: sentience in the broader sense. Recent controversies have focused on the sentience of fish and of invertebrates (including, but not limited to, octopuses). They have also tended to focus on sentience in the narrower sense: the capacity for valenced experience. What sort of evidence has been driving these debates?

The discussion of fish sentience has focussed primarily on the capacity for pain experience. Fish have been found to possess nociceptors (receptors specialized for the detection of noxious stimuli; Sneddon et al. (2003a)) that link to an integrative brain region, the telencephalon (Rink & Wulliman, 2004; Sneddon, 2004). A series of experiments on rainbow trout have found they display a range of physiological and behavioural responses to noxious stimuli, such as increased gill ventilation rate and performance of abnormal behaviours, including rubbing lips on the walls and floor of their tank after injection with acetic acid (Sneddon et al., 2003a). They also show evidence of change in motivational state, as fish exposed to noxious stimuli will feed less (Sneddon et al., 2003a), show lower levels of antipredator behaviour (Ashley et al., 2009) and reduced avoidance of unfamiliar objects (Sneddon et al., 2003b). These effects greatly diminish on treatment with the analgesic morphine, which acts in other animals to suppress the conscious experience of pain (Sneddon, 2003; Sneddon et al., 2003b). In combination with other evidence of complex perception, cognition, and behaviour (Brown, 2015), this has led many to conclude that there is sufficient evidence for pain experience and thus sentience in fish (Jones, 2013; Proctor, 2012; Sneddon et al., 2014).

One potential reason for caution here is that most of the tests described have been carried out on a small number of species (primarily rainbow trout and zebrafish), all belonging to the teleost group of fishes (infraclass Teleostei). What we commonly call ‘fish’ are not actually a single clade, but rather a paraphyletic grouping containing over 50 orders grouped into three separate classes: jawless fish such as hagfish and lampreys, cartilaginous fish such as sharks and rays, and bony fish such as teleosts. The distant relations between these groups weakens any generalisations we want to make between them. While almost all extant fish belong to the teleost group, this group still contains around 40 different orders and we must thus be careful about inferring too much about more distantly related species. Further work establishing similar physiology and behaviours in representatives of different orders would help strengthen the case for fishes more generally.

A very similar controversy has played out regarding invertebrates. Most work on sentience in invertebrates has been on three different groups: cephalopod molluscs, decapod crustaceans, and insects. Again, this work has tended to be pain‐centric. The complex behaviour and cognitive abilities of cephalopods (particularly octopuses) has led to widespread consensus regarding their sentience, as indicated by their mention in the Cambridge Declaration above, and which has led to their inclusion in animal protection legislation such as the EU Directive 2010/63/EU ‘on the protection of animals used for scientific purposes’, the UK's Animals (Scientific Procedures) Act 1986, that of two Australian states (Animal Welfare Act 1992 (ACT) and Animal Care and Protection Act 2001 (Qld)) and New Zealand's Animal Welfare Act 1999 (which also includes some decapod crustaceans).

Supporting this, there is a range of evidence for pain experience in cephalopods. Octopus and squid have been found to possess nociceptors, and these connect to the central nervous system (Alupay et al., 2014; Bazarini & Crook, 2020; Crook, 2021; Crook et al., 2013; Howard et al., 2019; Perez et al., 2017), though connection to the substantial integrative brain region of the vertical lobe is still to be established. Octopus and squid show behavioural changes after exposure to noxious stimuli, including defencive behaviour (Bazarini & Crook, 2020; Crook et al., 2011; Howard et al., 2019 **)**, increased responsiveness to threats (Crook et al., 2014; Oshima et al., 2016 **)**, and grooming injured arms (Alupay et al., 2014; Crook, 2021). Injured octopus have also been found to show a preference for a chamber containing analgesia (Crook, 2021). Much of this work is still quite new, but builds a growing picture supporting the consensus view on cephalopod sentience.

More controversial are decapod crustaceans: a diverse group of around 15,000 species, most of which have never been studied in relation to sentience (though the same could be said of vertebrates). Most research to date has focussed on crabs (both brachyuran and anomuran crabs) and astacid lobsters and crayfish. Decapod brains are complex, containing hemiellipsoid bodies that are thought to function as integrative centres (Sandeman et al., 2014). The presence of nociceptors has not yet been conclusively established, though there is evidence of the detection of extreme heat (Puri & Falks, 2010, 2015). Crabs and shrimp have shown some wound‐tending and rubbing behaviour (Barr et al., 2008; Diarte‐Plata et al., 2012; Dyuizen et al., 2012; Elwood et al., 2017; McCambridge et al., 2016), though another study failed to replicate this effect (Puri & Faulkes, 2010). Aside from pain, decapods display behaviours indicative of experiencing other affective states: when administered serotonin, crabs and crayfish show ‘anxiety‐like’ responses (Aggio et al., 1996, Fossat et al., 2014, 2015), and their anxiety‐like behaviour decreases if given fluoxetine (a selective serotonin uptake inhibitor; Hamilton et al. (2016)) or anxiolytics (Fossat et al., 2014, 2015). The ‘anxiety‐like’ behaviour in these studies is a reduction in exploratory behaviour, specifically a reluctance to enter the light arms of a maze in which some arms are light and others dark. There have also been studies of motivational trade‐offs in hermit crabs. When given electric shocks within a shell, hermit crabs are less likely to abandon the shell if it is a high‐quality shell (Appel & Elwood, 2009; Elwood & Appel, 2009).

A small amount of recent research has targeted the sentience of insects. Some insects, particularly bees, have demonstrated surprisingly complex behaviour and cognitive abilities (Chittka & Niven, 2009). The central complex of the insect brain is functionally similar to the vertebrate midbrain, leading Klein and Barron to argue, controversially, that insects are capable of subjective experience (Barron and Klein, 2016; Klein and Barron, 2016). This argument relies on the assumption that the vertebrate midbrain is sufficient for subjective experience, an idea powerfully advocated by Merker (2007) but also subject to significant criticism (Watkins & Rees, 2007).

Studies looking for markers of aversive experiences in insects have so far delivered mixed results. It has been reported that insects will continue normal feeding and mating behaviours even when severely injured (Eisemann et al., 1984), and there is evidence that injured bees will not preferentially self‐administer morphine when injured (Groening et al., 2017). This should be interpreted with caution, since there is no particular reason to expect morphine to be a good analgesic in insects (see Gibbons & Sarlak, 2020 for this and other criticisms). To further complicate the picture, earlier studies reported insects showing decreased behavioural response to noxious stimuli when given opioids, changes which were blocked by the opioid antagonist naloxone (Nunez et al., 1983; Zabala et al., 1984; Zabala & Gomez, 1991). We should be open to the possibility that the traditional focus on pain in this literature does not serve insects well. There are, of course, affects other than pain. For example, bees will show a ‘pessimistic’ judgement bias after being shaken (Bateson et al., 2011) and an ‘optimistic’ bias after being given an unexpected sugar reward (Perry et al., 2016), responses typically taken to be indicators of felt mood or welfare when found in vertebrates (Mendl et al., 2009).

Regarding the overall distribution of sentience, different reviews of the evidence have reached different conclusions. Jones (2013) concludes that some insects and crustaceans must feel pain, and the presence of innate analgesics in some snails and earthworms may also be suggestive of their capacity for pain. Broom (2016) considers that there is sufficient evidence for sentience in all vertebrates, cephalopods and decapod crustaceans, and thinks that cognitive ability in stomatopod crustaceans (mantis shrimp), spiders and some insects, including bees and ants, makes them good candidates for investigation. Ginsburg & Jablonka (2019, p. 351) attribute sentience to vertebrates, cephalopods, arthropods (insects and crustaceans) and possibly some annelids (worms). Godfrey‐Smith (2016) has also defended an inclusive view, writing that ‘the world is fuller, more replete with experience, than many people have countenanced’ (2020, p. 279). Others, however, are more sceptical.

Critics of attributions of sentience to fish and invertebrates (Key, 2016; Rose, 2002; Rose et al., 2014) point to the absence of a neocortex, the part of the brain that is most closely associated with conscious experience in humans. In humans, there is strong evidence for the involvement of the neocortex in the experience of pain, with the anterior cingulate cortex (or ACC) widely thought to play a particularly important role (Rainville et al., 1997). This leads to an argument that Dinets (2016) and Michel (2019) have called the ‘no cortex, no cry’ argument: a neocortex is necessary for pain, fish and invertebrates have no neocortex, therefore fish and invertebrates feel no pain.

As Michel notes, this argument is generally countered by an appeal to *multiple realizability*: we have no evidence that a neocortex is necessary for pain (or sentience more generally), because we have no evidence against the possibility that similar subjective experiences can be generated by very different mechanisms. The way subjective experience is implemented in one particular case need not be the only way.^3^ Indeed, we can conceive of the possibility of a conscious robot or AI system that feels pain without any neurons at all.

Yet, as Michel goes on to point out, it is not enough to appeal to multiple realizability and stop there. The appeal needs to be backed up with an explanation of why some systems are better candidates than others for being alternative realizations of sentience. We should concede that, in some cases, it is implausible to attribute pain solely on the basis of behaviours that, in humans, would be indicative of pain. Michel gives the example of the paramoecium^4^:

This unicellular organism covered with several thousand cilia can exhibit pain‐like behaviors: if a paramecium encounters a potentially dangerous concentrated salt solution or acetic acid, it will back away and swim in a different direction or engage in defensive behavior by discharging trichocysts. It could be that paramecia realize pain in a (yet) unknown way. (p. 2418)

Presumably, even proponents of fish sentience will grant the implausibility of attributing sentience to a single cell with no central nervous system. The absence of a central nervous system is intuitively a *defeater* for the inference from defencive behaviour to pain. But why is the absence of a central nervous system a defeater, whereas the absence of a neocortex is not? If we rely on inferring pain solely from behaviours that would indicate pain in humans, the burden lies on us to give a ‘theory of defeaters’ (Birch, 2020b) that can block this inference. Yet, at present, our theoretical understanding of the nature of consciousness is not mature enough to provide such a theory without bringing in highly controversial assumptions. This leaves us with a deep methodological problem.

## 3 MOVING FORWARD 1: PRECAUTIONARY REASONING ABOUT SENTIENCE

How can we move forward? Our solutions need to be sensitive to the context: the timescale on which an answer is needed, the purpose for which it will be used, the desired level of confidence, and what is at stake (cf. Birch, 2018; Douglas, 2009). In some contexts, precautionary reasoning (Steel, 2015) can help with questions about sentience (Birch, 2017; Bradshaw, 1998; Knutsson and Munthe, 2017). The case is particularly compelling when we are faced with the question of how to set the scope of animal welfare law.

Consider the question: should decapod crustaceans be protected by animal welfare law? A lot is at stake, because crustaceans are caught from the wild and farmed on a massive scale. It has been estimated that at least 250 billion decapod crustaceans are farmed each year—the number caught from the wild is more or less impossible to estimate reliably, but is likely to be much larger.^5^ We can identify some clear welfare risks. Some of the practices to which decapods are exposed (including declawing, live boiling, and osmotic shock) are likely to cause great suffering if the animals are sentient.

This is a context in which there is an asymmetry of risk. The risks of incorrectly regarding decapods as non‐sentient when they are sentient are severe: the main risk is that we will continue to fail to regulate practices that are causing immense suffering to billions of animals. On the other side of the ledger, the risks of incorrectly regarding decapods as sentient when they are non‐sentient are plausibly much less severe. While there is admittedly a risk of some businesses suffering from over‐regulation, there is a corresponding opportunity for businesses that already follow good‐practice guidance and already take precautions to benefit from seeing their high standards properly enforced.

Bradshaw (1998) has even suggested that, given this asymmetry of risk, we should assume *all* animals to be sentient, with or without evidence. If we add the qualification *in a policy‐making context*, that proposal may not be as extreme as it initially sounds. In a policy‐making context, there are advantages to skipping the vexed question of sentience, at least initially, in order to move straight to the question of what welfare risks exist *if* the animals are sentient, and what precautions would be feasible and proportionate in relation to those risks. The evidence regarding sentience would then inform the design of precautions, rather than providing a trigger for precautions to be considered at all. For example, there is a great deal of evidence regarding more and less humane slaughter methods in mammals, making specific and detailed regulations possible. A comparable evidence base for insects simply does not exist, partly because our understanding of sentience in insects is so limited. A better evidence base would allow for evidence‐based precautions.

Birch’s (2017) ‘animal sentience precautionary principle’ takes a somewhat less generous approach to precautionary attributions of sentience, requiring that at least one ‘credible indicator’ from a consensus list needs to be present before the bar for precautionary action is cleared. Some invertebrates (cephalopod molluscs, decapod crustaceans) clear that bar, for reasons discussed in the previous section, but many would not. When it comes to insects, the question of whether they clear the bar will depend on which credible indicators make it on to the list. Some critics of Birch's proposal (Brown, 2017; Mallatt, 2017; Woodruff, 2017) have argued for setting a higher bar, involving at least two credible indicators obtained independently of each other. But the general idea of taking a precautionary approach to attributing sentience in policy‐making contexts appears to command widespread support (though see Diggles, 2019 for dissent).

## 4 MOVING FORWARD 2: STUDYING SENTIENCE USING METHODS FROM HUMAN CONSCIOUSNESS SCIENCE

Precautionary reasoning is a tool for policy‐making. Scientists too sometimes set policies (e.g. rules for their own labs), and in these contexts, the same case for precautionary attributions of sentience can be made. However, it would be a confusion to suggest that precautionary reasoning can settle the scientific question of which animals are sentient. It cannot. The scientific question remains substantially open at present, for the reasons given by Dawkins, Michel and others. Since the source of the trouble is our limited understanding of phenomenal consciousness, the question is open for both the broader and narrower senses of sentience.

How might the scientific question be settled? One way or another, the answer must be to take inspiration from the science of human consciousness, which has developed a battery of methods for probing the neural and cognitive signatures of conscious experience. But there is a big problem here: these methods still rely heavily on a subject's ability to report their experiences, and the ability of non‐human animals to report their experiences is at best very limited (Boly et al., 2013; LeDoux, 2019). Translating the exact same methods across to non‐human animals will not work. Moreover, we cannot safely assume that ‘no report’ indicators developed for humans (such as the use of eye movements and pupil size to detect binocular rivalry; Tsuchiya et al., 2015, Pitts et al., 2018), even if valid in the human case, are still tracking conscious experience in non‐human animals (Browning & Veit, 2020). How is this problem to be overcome?

One strategy would be to wait for the science of human consciousness to converge on an empirically well‐confirmed, general theory of consciousness, and then simply *apply* that theory to non‐human animals. Some reasons for scepticism about this strategy can be brought out by considering one particularly influential theory: the global neuronal workspace (GNW) theory (Dehaene, 2014; Dehaene et al., 2011; Mashour et al., 2020). The GNW theory posits the existence in the brain of a global broadcast mechanism that integrates representations from perceptual systems, affective systems, and memory systems and broadcasts the integrated content back to the input systems and onward to a wide variety of consumer systems, including mechanisms of voluntary report, planning, reasoning, and decision making (Dehaene & Changeux, 2011, p. 209). The representations currently being broadcast are said to be in the global workspace. Interpreted as a theory of phenomenal consciousness and not just cognitive access, the theory states that a representation becomes phenomenally conscious when it enters the workspace, whereas more localized processing outside the workspace occurs without phenomenal consciousness.

Suppose this theory is true: what does it say about animals? In humans, the GNW is dependent on the neocortex. As a result, there is no chance that we will find the human GNW in its entirety in a fish brain, an invertebrate brain, or even (for that matter) in the brain of a bird (Bugnyar and Güntürkün, 2016). What we may well find are substantially different mechanisms realizing a functionally similar workspace. As Dehaene has put it, ‘I would not be surprised if we discovered that all mammals, and probably many species of birds and fish, show evidence of a convergent evolution to the same sort of conscious workspace’ (Dehaene, 2014, p. 246). Some tentative evidence of such a workspace in crows is provided by Nieder et al. (2020).

We will then face a version of the old multiple realization controversy, introduced earlier in the case of pain, but this time regarding conscious experience in general. Exactly how similar to the human global workspace does a workspace have to be to suffice for conscious experience? How different is too different? The GNW theory as currently formulated is silent on this question. Moreover, it is hard to see how any amount of evidence *from humans* could allow a principled answer, and no less hard to see how we could answer it using evidence *from animals* without independently knowing which animals are sentient and which are not. Carruthers (2018a,b, 2019, 2020) has suggested that, since the GNW theory is plausibly true and yet unable to settle questions about animal sentience, we should accept there is no fact of the matter about those questions ‐ and stop asking them.^6^ We draw a different moral: we need new methodological strategies for investigating animal sentience that look beyond the GNW theory, and indeed beyond any currently fashionable theory.

One way forward, suggested by Birch (2020b), is based on the ‘facilitation hypothesis’: the hypothesis that conscious perception of a stimulus *facilitates*, relative to unconscious perception, a cluster of cognitive abilities in relation to that stimulus. The facilitation hypothesis is a piece of theorizing about the relationship between cognition and conscious experience, but it is more general than any current theory of consciousness. Indeed, Birch argues that it is compatible with all the main current theories, and receives support from a wide range of empirical studies that show (for some particular ability) that humans perform better in tests of that ability when a stimulus is perceived consciously rather than unconsciously.

To give one example: trace conditioning is a form of classical conditioning in which the two stimuli are separated by a temporal interval. For example, a tone in your ear may be followed, one second later, by a puff of air aimed at your eye. This form of learning has long been linked to conscious perception (Clark & Squire, 1998; Clark et al., 2002). This points towards conscious experience playing a role in facilitating temporal cognition and the learning of temporal relations, and several authors have suggested this may offer a window into conscious perception in non‐human animals (Allen, 2004, 2013; Birch, 2020b; Birch et al., 2020a; Droege et al., 2021).

Finding trace conditioning in an animal would not, by itself, be very strong evidence of sentience (even in the broader sense), because it may be that other animals can learn temporal relations without conscious perception. For example, evidence of olfactory trace conditioning in honey bees (Szyszka et al., 2011) does not, by itself, make a strong case for sentience in honey bees. But trace conditioning is just one example of an ability facilitated by conscious perception in humans. Other examples include rapid reversal learning (Travers et al., 2018) and instrumental conditioning (Skora et al., 2021), and we can expect the science of human consciousness to find more candidates as it progresses. If evidence of trace conditioning were to be augmented by evidence of another ability facilitated by conscious perception in humans, and another, and another… a large and diverse cluster of abilities would provide a platform for asking whether there is a distinctive kind of processing that, in the animal in question, facilitates *all* of these abilities, in the way that conscious perception does in humans. That distinctive kind of processing would then be a strong candidate for the neural basis of conscious perception in the species in question.

This is one example of an approach to animal sentience that explores the middle ground between committing to a specific theory that extrapolates speculatively from the human case and attempting to proceed in a completely theory‐free way. Other explorations of this middle ground include the ‘natural kind approach’ (Bayne, 2018; Shea, 2012; Shea and Bayne, 2010), the ‘Unlimited Associative Learning’ approach (Birch et al., 2020a; Bronfman et al., 2016; Ginsburg and Jablonka, 2019) and Feinberg and Mallatt's (2016, 2018, 2019) approach based on multisensory maps. We cannot explain all of these approaches here, but it is worth noting that they all rely on a *minimal* theoretical commitment about the relation between sentience (typically in the broader sense) and cognition ‐ and use this as the organizing principle of an empirical research programme that may, in the long run, help us build better, more complete theories. Birch (2020b) calls this general methodological strategy the ‘theory‐light’ strategy.

When drawing inspiration from human consciousness science, it is natural to look first at conscious *perception* ‐ and specifically conscious *vision* ‐ because it is in the case of vision that we have well‐validated methods for disentangling conscious and unconscious processing in the human case. One example is backward masking, in which a stimulus is rendered subliminal by presenting another stimulus immediately afterwards (Dehaene, 2014). Translating paradigms such as backward masking to animals raises new complications, of course, but there is no reason to think the challenges insurmountable. Indeed, Ben‐Haim et al. (2021) recently used backward masking to dissociate two types of visual processing in macaques. By contrast, we have a much weaker grip on how to disentangle conscious and unconscious *affect*. There is no equivalent of backward masking for affect. Yet it is conscious affect that matters most directly to animal welfare. We see this as a largely unsolved problem and an important research priority (see also Paul et al., 2020).

## 5 CONCLUSION

The interdisciplinary field of animal sentience research is beginning to take off (see, in particular, the journal *Animal Sentience*, founded in 2016). This is good news, because, as our brief overview has shown, there are many open questions (scientific, epistemological, metaphysical, ethical, political) in need of further work. These include:

1. How can we disentangle conscious and unconscious affect in non‐human animals?
2. How can we translate methods from human consciousness science to animal sentience research?
3. How can we draw on evidence from animals to build better theories of the fundamental nature of conscious experience?
4. How can we understand the variation in the richness and structure of subjective experiences across the animal kingdom?^7^
5. In the face of uncertain evidence, what precautionary measures are proportionate for protecting the welfare of potentially sentient animals?

We encourage all readers of this article, whatever their background, to take up the search for answers.

## CONFLICT OF INTEREST

The authors declare that there are no conflicts of interest.

## ENDNOTES

^1^
For an attempt to define ‘phenomenal consciousness’ by example, see Schwitzgebel (2016). For empirical investigations of the ordinary concept of ‘subjective experience’, see Sytsma and Machery (2010), Sytsma (2014). Sytsma and Machery (2010) offer some evidence in support of the idea that the ordinary concept implies valence, bringing it closer to our ‘narrower’ sense of the concept.

^2^
Some authors add additional requirements, leading to yet narrower senses of the word. For example, Broom (2014), a leading animal welfare scientist, writes that ‘a sentient being is one that has some ability (i) to evaluate the actions of others in relation to itself and third parties, (ii) to remember some of its actions and their consequences, (iii) to assess risks and benefits, (iv) to have some feelings and (v) to have some degree of awareness’ (2014, p. 5).

^3^
Versions of this point were made by many commentators on Key (2016), by Tye (2017), and more recently by Browning and Veit (2020).

^4^
Michel's paper also includes a discussion of pain‐like behaviour in decapitated frogs. Allen (2004) and Allen et al. (2009) discuss the example of pain‐like behaviour controlled by spinal cords disconnected from the brain.

^5^

http://fishcount.org.uk/fish‐count‐estimates‐2/numbers‐of‐farmed‐decapod‐crustaceans.

^6^
Carruthers' arguments are complex and we cannot do them justice here. See Black (2020), Birch (2020a) and Simons (2020) for criticism.

^7^
This topic is introduced by Birch et al. (2020b).

## References

[phc312822-bib-0001] Aggio, J. , Ratikin, A. , & Maldonado, H. (1996). Serotonin‐induced short‐ and long‐term sensitization in the crab Chasmagnathus. Pharmacology, Biochemistry and Behavior, 53(2), 441–448. 10.1016/0091-3057(95)02015-2 8808156

[phc312822-bib-0002] Allen, C. (2004). Animal pain. Noûs, 38(4), 614–643. 10.1111/j.0029-4624.2004.00486.x

[phc312822-bib-0003] Allen, C. (2013). Fish cognition and consciousness. Journal of Agricultural and Environmental Ethics, 26(1), 25–39. 10.1007/s10806-011-9364-9

[phc312822-bib-0004] Allen, C. , Grau, J. W. , & Meagher, M. W. (2009). The lower bounds of cognition: What do spinal cords reveal? In J. Bickle (Ed.), The Oxford handbook of philosophy and neuroscience (pp. 129–142). Oxford University Press.

[phc312822-bib-0005] Alupay, J. S. , Hadjisolomou, S. P. , & Crook, R. J. (2014). Arm injury produces long‐term behavioral and neural hypersensitivity in octopus. Neuroscience Letters, 558, 137–142. 10.1016/j.neulet.2013.11.002 24239646

[phc312822-bib-0006] Appel, M. , & Elwood, R. W. (2009). Motivational trade‐offs and potential pain experience in hermit crabs. Applied Animal Behaviour Science, 119(1–2), 120–124. 10.1016/j.applanim.2009.03.013

[phc312822-bib-0007] Ashley, P. J. , Ringrose, S. , Edwards, K. L. , Wallington, E. , McCrohan, C. R. , & Sneddon, L. U. (2009). Effect of noxious stimulation upon antipredator responses and dominance status in rainbow trout. Animal Behaviour, 77(2), 403–410. 10.1016/j.anbehav.2008.10.015

[phc312822-bib-0008] Auvray, M. , Myin, E. , & Spence, C. (2010). The sensory‐discriminative and affective‐ motivational aspects of pain. Neuroscience & Biobehavioral Reviews, 34(2), 214–223. 10.1016/j.neubiorev.2008.07.008 18718486

[phc312822-bib-0009] Barr, S. , Laming, P. R. , Dick, J. T. , & Elwood, R. W. (2008). Nociception or pain in a decapod crustacean? Animal Behaviour, 75(3), 745–751. 10.1016/j.anbehav.2007.07.004

[phc312822-bib-0134] Barron, A. B. , & Klein, C. (2016). What insects can tell us about the origins of consciousness. Proceedings of the National Academy of Sciences, 113(18), 4900–4908. 10.1073/pnas.1520084113 PMC4983823 27091981

[phc312822-bib-0010] Bateson, M. , Desire, S. , Gartside, S. E. , & Wright, G. A. (2011). Agitated honeybees exhibit pessimistic cognitive biases. Current Biology, 21(12), 1070–1073. 10.1016/j.cub.2011.05.017 21636277 PMC3158593

[phc312822-bib-0011] Bayne, T. (2018). On the axiomatic foundations of the integrated information theory of consciousness. Neuroscience of consciousness, 4(1), niy007. 10.1093/nc/niy007 PMC6030813 30042860

[phc312822-bib-0012] Bazarini, S. N. , & Crook, R. J. (2020). Environmental estrogen exposure disrupts sensory processing and nociceptive plasticity in the cephalopod Euprymna scolopes. Journal of Experimental Biology, 223, jeb218008. 10.1242/jeb.218008 32487666

[phc312822-bib-0013] Ben‐Haim, M. S. , Dal Monte, O. , Fagan, N. A. , Dunham, Y. , Hassin, R. R. , Chang, S. W. , & Santos, L. R. (2021). Disentangling perceptual awareness from nonconscious processing in rhesus monkeys (Macaca mulatta). Proceedings of the National Academy of Sciences, 118(15), e2017543118. 10.1073/pnas.2017543118 PMC8053918 33785543

[phc312822-bib-0014] Bentham, J. (1789). An introduction to the principles of morals and legislation. T. Payne and son.

[phc312822-bib-0015] Berridge, K. , & Winkielman, P. (2003). What is an unconscious emotion? (The case for unconscious “liking”). Cognition & Emotion, 17(2), 181–211. 10.1080/02699930302289 29715719

[phc312822-bib-0016] Birch, J. (2017). Animal sentience and the precautionary principle. Animal Sentience, 2(16), 1. 10.51291/2377-7478.1200. https://animalstudiesrepository.org/animsent/vol2/iss16/1/

[phc312822-bib-0017] Birch, J. (2018). Animal cognition and human values. Philosophy of Science, 85(5), 1026–1037. 10.1086/699744

[phc312822-bib-0020] Birch, J. (2020a). Global workspace theory and animal consciousness. Philosophical Topics, 48(1), 21–38. http://philsci‐archive.pitt.edu/18031/

[phc312822-bib-0018] Birch, J. (2020b). The search for invertebrate consciousness. Noûs. Advance online publication. 10.1111/nous.12351 PMC7612530 35321054

[phc312822-bib-0019] Birch, J. , Ginsburg, S. , & Jablonka, E. (2020a). Unlimited Associative Learning and the origins of consciousness: a primer and some predictions. Biology and Philosophy, 35(6), 56. 10.1007/s10539-020-09772-0 33597791 PMC7116763

[phc312822-bib-0021] Birch, J. , Schnell, A. K. , & Clayton, N. S. (2020b). Dimensions of animal consciousness. Trends in Cognitive Sciences, 24(10), 789–801. 10.1016/j.tics.2020.07.007 32830051 PMC7116194

[phc312822-bib-0022] Black, D. (2020). The global workspace theory, the phenomenal concept strategy, and the distribution of consciousness. Consciousness and Cognition, 84, 102992. 10.1016/j.concog.2020.102992 32771955

[phc312822-bib-0023] Block, N. J. (1995). On a confusion about the function of consciousness. Behavioral and Brain Sciences, 18(2), 227–247. 10.1017/s0140525x00038188

[phc312822-bib-0024] Boly, M. , Seth, A. K. , Wilke, M. , Ingmundson, P. , Baars, B. , Laureys, S. , Edelman, D. , & Tsuchiya, N. (2013). Consciousness in humans and non‐human animals: Recent advances and future directions. Frontiers in Psychology, 4, 625. 10.3389/fpsyg.2013.00625 24198791 PMC3814086

[phc312822-bib-0025] Bradshaw, R. H. (1998). Consciousness in non‐human animals: adopting the precautionary principle. Journal of Consciousness Studies, 5, 108–114.

[phc312822-bib-0026] Bronfman, Z. Z. , Ginsburg, S. , & Jablonka, E. (2016). The transition to minimal consciousness through the evolution of associative learning. Frontiers in Psychology, 7, 1954. 10.3389/fpsyg.2016.01954 28066282 PMC5177968

[phc312822-bib-0027] Broom, D. M. (2014). Sentience and animal welfare. CABI.

[phc312822-bib-0028] Broom, D. M. (2016). Considering animals feelings. Animal Sentience, 1, 1.

[phc312822-bib-0029] Brown, C. (2015). Fish intelligence, sentience and ethics. Animal Cognition, 18, 1–17. 10.1007/s10071-014-0761-0 24942105

[phc312822-bib-0030] Brown, C. (2017). A risk assessment and phylogenetic approach. Animal Sentience, 16, 3. 10.51291/2377-7478.1219

[phc312822-bib-0031] Browning, H. (2020). If I could talk to the animals: Measuring subjective animal welfare. Ph.D. thesis. Australian National University. 10.25911/5f1572fb1b5be

[phc312822-bib-0032] Browning, H. , & Veit, W. (2020). The measurement problem of consciousness. Philosophical Topics, 48(1), 85–108.

[phc312822-bib-0033] Carruthers, P. (2018a). Comparative psychology without consciousness. Consciousness and Cognition, 63, 47–60. 10.1016/j.concog.2018.06.012 29940429

[phc312822-bib-0034] Carruthers, P. (2018b). The problem of animal consciousness. Proceedings and Addresses of the American Philosophical Association, 92, 179–205.

[phc312822-bib-0035] Carruthers, P. (2019). Human and Animal Minds: The Consciousness Questions Laid to Rest. Oxford University Press.

[phc312822-bib-0036] Carruthers, P. (2020). Stop caring about consciousness. Philosophical Topics, 48(1), 1–20. 10.5840/philtopics20204811

[phc312822-bib-0037] Chittka, L. , & Niven, J. (2009). Are bigger brains better? Current Biology, 19(21), R995–R1008. 10.1016/j.cub.2009.08.023 19922859

[phc312822-bib-0038] Clark, R. E. , Manns, J. R. , & Squire, L. H. (2002). Classical conditioning, awareness, and brain systems. Trends in Cognitive Sciences, 12, 524–531. 10.1016/s1364-6613(02)02041-7 12475713

[phc312822-bib-0039] Clark, R. E. , & Squire, L. R. (1998). Classical conditioning and brain systems: The role of awareness. Science, 280(5360), 77–81. 10.1126/science.280.5360.77 9525860

[phc312822-bib-0040] Crook, R. J . (2021). Behavioral and neurophysiological evidence suggests affective pain experience in octopus. iScience, 24(3), 102229. 10.1016/j.isci.2021.102229 33733076 PMC7941037

[phc312822-bib-0041] Crook, R. J. , Dickson, K. , Hanlon, R. T. , & Walters, E. T. (2014). Nociceptive sensitization reduces predation risk. Current Biology, 24(10), 1121–1125. 10.1016/j.cub.2014.03.043 24814149

[phc312822-bib-0042] Crook, R. J. , Hanlon, R. T. , & Walters, E. T. (2013). Squid have nociceptors that display widespread long‐term sensitization and spontaneous activity after bodily injury. Journal of Neuroscience, 33(24), 10021–10026. 10.1523/jneurosci.0646-13.2013 23761897 PMC4767780

[phc312822-bib-0043] Crook, R. J. , Lewis, T. , Hanlon, R. T. , & Walters, E. T. (2011). Peripheral injury induces long‐ term sensitization of defensive responses to visual and tactile stimuli in the squid, Loligo pealeii. Journal of Experimental Biology, 24, 3171–3185. 10.1242/jeb.058131 PMC3168376 21900465

[phc312822-bib-0044] Dawkins, M. S. (2012). Why animals matter: Animal consciousness, animal welfare and human well‐being. Oxford University Press.

[phc312822-bib-0045] Dawkins, M. S. (2017). Animal welfare with and without consciousness. Journal of Zoology, 301, 1–10. 10.1111/jzo.12434

[phc312822-bib-0046] Dawkins, M. S. (2021). The science of animal welfare: Understanding what animals want. Oxford University Press.

[phc312822-bib-0047] DeGrazia, D. (1996). Taking animals seriously: Mental life and moral status. Cambridge University Press.

[phc312822-bib-0048] Dehaene, S. (2014). Consciousness and the brain: Deciphering how the brain encodes our thoughts. Viking Press.

[phc312822-bib-0049] Dehaene, S. , & Changeux, J.‐P. (2011). Experimental and theoretical approaches to conscious processing. Neuron, 70(2), 200–227. 10.1016/j.neuron.2011.03.018 21521609

[phc312822-bib-0050] Dehaene, S. , Changeux, J. P. , & Naccache, L. (2011). The global neuronal workspace model of conscious access: From neuronal architectures to clinical applications. In S. Dehaene & Y. Christen (Eds.), Characterizing Consciousness: From Cognition to the Clinic? (pp. 55–84). Springer.

[phc312822-bib-0051] Diarte‐Plata, G. , Sainz‐Hernández, J. C. , Aguinãga‐Cruz, J. A. , Fierro‐Coronado, J. A. , Polanco‐Torres, A. , & Puente‐Palazuelos, C. (2012). Eyestalk ablation procedures to minimize pain in the freshwater prawn Macrobrachium americanum. Applied Animal Behaviour Science, 140(3–4), 172–178. 10.1016/j.applanim.2012.06.002

[phc312822-bib-0052] Diggles, B. K. (2019). Review of some scientific issues related to crustacean welfare. ICES Journal of Marine Science, 76(1), 66–81. 10.1093/icesjms/fsy058

[phc312822-bib-0053] Dinets, V. (2016). No cortex, no cry. Animal Sentience, 1(3), 7. 10.51291/2377-7478.1027

[phc312822-bib-0054] Douglas, H. E. (2009). Science, Policy and the Value‐Free Ideal. University of Pittsburgh Press.

[phc312822-bib-0055] Droege, P. , Weiss, D. J. , Schwob, N. , & Braithwaite, V. (2021). Trace conditioning as a test for animal consciousness: a new approach. Animal Cognition. Advance online publication, 10.1007/s10071-021-01522-3 33983542

[phc312822-bib-0056] Duncan, I. J. (2002). Poultry welfare: Science or subjectivity? British Poultry Science, 43(5), 643–652. 10.1080/0007166021000025109 12555888

[phc312822-bib-0057] Duncan, I. J. (2006). The changing concept of animal sentience. Applied Animal Behaviour Science, 100(1–2), 11–19. 10.1016/j.applanim.2006.04.011

[phc312822-bib-0058] Dyuizen, I. V. , Kotsyuba, E. P. , & Lamash, N. E. (2012). Changes in the nitric oxide system in the shore crab Hemigrapsus sanguineus (Crustacea, Decapoda) CNS induced by a nociceptive stimulus. Journal of Experimental Biology, 215(15), 2668–2676. 10.1242/jeb.066845 22786644

[phc312822-bib-0059] Eisemann, C. H. , Jorgensen, W. K. , Merritt, D. J. , Rice, M. J. , Cribb, B. W. , Webb, P. D. , & Zalucki, M. P. (1984). Do insects feel pain?—A biological view. Experientia, 40(2), 164–167. 10.1007/bf01963580

[phc312822-bib-0060] Elwood, R. W. , & Appel, M. (2009). Pain experience in hermit crabs? Animal Behaviour, 77(5), 1243–1246. 10.1016/j.anbehav.2009.01.028

[phc312822-bib-0061] Elwood, R. W. , Dalton, N. , & Riddell, G. (2017). Aversive responses by shore crabs to acetic acid but not to capsaicin. Behavioural Processes, 140, 1–5. 10.1016/j.beproc.2017.03.022 28365460

[phc312822-bib-0062] Feinberg, T. E. , & Mallatt, J. (2019). Subjectivity “demystified”: Neurobiology, evolution, and the explanatory gap. Frontiers in Psychology, 10, 1686. 10.3389/fpsyg.2019.01686 31417451 PMC6685416

[phc312822-bib-0063] Feinberg, T. E. , & Mallatt, J. M. (2016). The ancient origins of consciousness: How the brain created experience. MIT Press.

[phc312822-bib-0064] Feinberg, T. E. , & Mallatt, J. M. (2018). Consciousness demystified. MIT Press.

[phc312822-bib-0065] Fossat, P. , Bacqué‐Cazenave, J. , De Deurwaerdère, P. , Cattaert, D. , & Delbecque, J. P. (2015). Serotonin, but not dopamine, controls the stress response and anxiety‐like behavior in the crayfish Procambarus clarkii. Journal of Experimental Biology, 218, 2745–2752. 10.1242/jeb.120550 26139659

[phc312822-bib-0066] Fossat, P. , Bacqué‐Cazenave, J. , De Deurwaerdère, P. , Delbecque, J. P. , & Cattaert, D. (2014). Anxiety‐like behavior in crayfish is controlled by serotonin. Science, 344(6189), 1293–1297. 10.1126/science.1248811 24926022

[phc312822-bib-0067] Gibbons, M. , & Sarlak, S. (2020). Inhibition of pain or response to injury in invertebrates and vertebrates. Animal Sentience, 29, 34. 10.51291/2377-7478.1649

[phc312822-bib-0068] Ginsburg, S. , & Jablonka, E. (2019). The evolution of the sensitive soul: Learning and the origins of consciousness. MIT Press.

[phc312822-bib-0069] Godfrey‐Smith, P. (2016). Other minds: The octopus, the sea, and the deep origins of consciousness. Farrar, Strauss and Giroux.

[phc312822-bib-0070] Goodwin, G. P. (2015). Experimental approaches to moral standing. Philosophy Compass, 10(12), 914–926. 10.1111/phc3.12266

[phc312822-bib-0071] Groening, J. , Venini, D. , & Srinivasan, M. (2017). In search of evidence for the experience of pain in honeybees: A self‐administration study. Scientific Reports, 7(1), 45825. 10.1038/srep45825 28374827 PMC5379194

[phc312822-bib-0072] Güntürkün, O. , & Bugnyar, T. (2016). Cognition without cortex. Trends in Cognitive Sciences, 20(4), 291–303. 10.1016/j.tics.2016.02.001 26944218

[phc312822-bib-0073] Hamilton, T. J. , Kwan, G. T. , Gallup, J. , & Tresguerres, M. (2016). Acute fluoxetine exposure alters crab anxiety‐like behaviour, but not aggressiveness. Scientific Reports, 6(1), 19850. 10.1038/srep19850 26806870 PMC4726416

[phc312822-bib-0074] Harnad, S. (2016). Animal sentience: the other minds problem. Animal Sentience, 1(1). 10.51291/2377-7478.1065. https://www.wellbeingintlstudiesrepository.org/animsent/vol1/iss1/1/

[phc312822-bib-0075] Howard, R. B. , Lopes, L. N. , Lardie, C. R. , Perez, P. P. , & Crook, R. J. (2019). Early‐life injury produces lifelong neural hyperexcitability, cognitive deficit and altered defensive behaviour in the squid Euprymna scolopes. Philosophical Transactions of the Royal Society of London B Biological Sciences, 374(1785), 20190281. 10.1098/rstb.2019.0281 31544621 PMC6790388

[phc312822-bib-0076] Jack, A. I. , & Robbin, P. (2012). The phenomenal stance revisited. Review of Philosophy and Psychology, 3, 383–403. 10.1007/s13164-012-0104-5

[phc312822-bib-0077] Jones, R. C. (2013). Science, sentience, and animal welfare. Biology and Philosophy, 28, 1–30. 10.1007/s10539-012-9351-1

[phc312822-bib-0078] Key, B. (2016). Why fish do not feel pain. Animal Sentience, 3, 1. 10.51291/2377-7478.1011

[phc312822-bib-0079] Kirkwood, J. K. (2006). The distribution of the capacity for sentience in the animal kingdom. In J. Turner & J. D’Silva (Eds.), Animals, Ethics and Trade: The Challenge of Animal Sentience (pp. 12–26). Compassion in World Farming Trust.

[phc312822-bib-0080] Klein, C. , & Barron, A. B. (2016). Insects have the capacity for subjective experience. Animal Sentience, 9, 1. 10.51291/2377-7478.1113

[phc312822-bib-0081] Knutsson, S. , & Munthe, C. (2017). A virtue of precaution regarding the moral status of animals with uncertain sentience. Journal of Agricultural and Environmental Ethics, 30(2), 213–224. 10.1007/s10806-017-9662-y

[phc312822-bib-0082] LeDoux, J. (2019). The deep history of ourselves: The four‐billion‐year story of how we got conscious brains. Viking Press.

[phc312822-bib-0083] Low, P. , Panksepp, J. , Reiss, D. , Edelman, D. , van Swinderen, B. , & Koch, C. (2012). The Cambridge Declaration on Consciousness. Retrieved from https://fcmconference.org/img/CambridgeDeclarationOnConsciousness.pdf

[phc312822-bib-0084] Mallatt, J. (2017). Shoring up the precautionary BAR. Animal Sentience, 16, 7. 10.51291/2377-7478.1229

[phc312822-bib-0085] Mashour, G. A. , Roelfsema, P. , Changeux, J. P. , & Dehaene, S. (2020). Conscious processing and the global neuronal workspace hypothesis. Neuron, 105(5), 776–798. 10.1016/j.neuron.2020.01.026 32135090 PMC8770991

[phc312822-bib-0086] McCambridge, C. , Dick, J. T. , & Elwood, R. W. (2016). Effects of autotomy compared to manual declawing on contests between males for females in the edible crab cancer pagurus: Implications for fishery practice and animal welfare. Journal of Shellfish Research, 35(4), 1037–1044. 10.2983/035.035.0426

[phc312822-bib-0087] Mellor, D. J. , Beausoleil, N. J. , Littlewood, K. E. , McLean, A. N. , McGreevy, P. D. , Jones, B. , & Wilkins, C. (2020). The 2020 Five Domains model: Including human–animal interactions in assessments of animal welfare. Animals, 10, 1870. 10.3390/ani10101870 PMC7602120 33066335

[phc312822-bib-0088] Mendl, M. , Burman, O. H. P. , Parker, R. M. A. , & Paul, E. S. (2009). Cognitive bias as an indicator of animal emotion and welfare: Emerging evidence and underlying mechanisms. Applied Animal Behaviour Science, 118(3–4), 161–181. 10.1016/j.applanim.2009.02.023

[phc312822-bib-0089] Merker, B. H . (2007). Consciousness without a cerebral cortex: A challenge for neuroscience and medicine. Behavioral and Brain Sciences, 30, 63–81. 10.1016/S1053-8100(03)00002-3 17475053

[phc312822-bib-0090] Michel, M. (2019). Fish and microchips: On fish pain and multiple realization. Philosophical Studies, 25(9), 95–110. 10.1007/s11098-018-1133-4

[phc312822-bib-0091] Nagel, T. (1974). What is it like to be a bat? Philosophical Review. Philosophical Review, 83(4), 435–450. 10.2307/2183914

[phc312822-bib-0092] Nieder, A. , Wagener, L. , & Rinnert, P. (2020). A neural correlate of sensory consciousness in a corvid bird. Science, 369(6511), 1626–1629. 10.1126/science.abb1447 32973028

[phc312822-bib-0093] Núñez, J. , Maldonado, H. , Miralto, A. , & Balderrama, N. (1983). The stinging response of the honeybee: Effects of morphine, naloxone and some opioid peptides. Pharmacology Biochemistry and Behavior, 19(6), 921–924. 10.1016/0091-3057(83)90391-x 6657718

[phc312822-bib-0094] Oshima, M. , di Pauli von Treuheim, T. , Carroll, J. , Hanlon, R. T. , Walters, E. T. , & Crook, R. J. (2016). Peripheral injury alters schooling behavior in squid, Doryteuthis pealeii. Behavioural Processes, 128, 89–95. 10.1016/j.beproc.2016.04.008 27108689

[phc312822-bib-0095] Paul, E. S. , Sher, S. , Tamietto, M. , Winkielman, P. , & Mendl, M. T. (2020). Towards a comparative science of emotion: Affect and consciousness in humans and animals. Neuroscience & Biobehavioral Reviews, 108, 749–770. 10.1016/j.neubiorev.2019.11.014 31778680 PMC6966324

[phc312822-bib-0096] Perez, P. V. , Butler‐Struben, H. M. , & Crook, R. J. (2017). The selective serotonin reuptake inhibitor fluoxetine increases spontaneous afferent firing, but not mechanonociceptive sensitization, in octopus. Invertebrate Neuroscience, 17(4), 10. 10.1007/s10158-017-0203-1 28988319

[phc312822-bib-0097] Perry, C. J. , Baciadonna, L. , & Chittka, L. (2016). Unexpected rewards induce dopamine‐dependent positive emotion–like state changes in bumblebees. Science, 353(6307), 1529–1531. 10.1126/science.aaf4454 27708101

[phc312822-bib-0098] Pitts, M. A. , Lutsyshyna, L. A. , & Hillyard, S. A. (2018). The relationship between attention and consciousness: an expanded taxonomy and implications for ‘no‐report’ paradigms. Philosophical Transactions of the Royal Society B: Biological Sciences, 373(1755), 20170348. 10.1098/rstb.2017.0348 PMC6074089 30061462

[phc312822-bib-0099] Powell, R. , & Mikhalevich, I. (2021). Affective sentience and moral protection. Animal Sentience, 29. 10.51291/2377-7478.1668

[phc312822-bib-0100] Proctor, H. (2012). Animal sentience: Where are we and where are we heading? Animals, 2(4), 628–639. 10.3390/ani2040628 26487167 PMC4494284

[phc312822-bib-0101] Puri, S. , & Faulkes, Z. (2010). Do decapod crustaceans have nociceptors for extreme pH? PLoS One, 5(4), e10244. 10.1371/journal.pone.0010244 20422026 PMC2857684

[phc312822-bib-0102] Puri, S. , & Faulkes, Z. (2015). Can crayfish take the heat? Procambarus clarkii show nociceptive behaviour to high temperature stimuli, but not low temperature or chemical stimuli. Biology Open, 4, 441–448. 10.1242/bio.20149654 25819841 PMC4400587

[phc312822-bib-0103] Rainville, P. , Duncan, G. H. , Price, D. D. , Carrier, B. , & Bushnell, M. C. (1997). Pain affect encoded in human anterior cingulate but not somatosensory cortex. Science, 277(5328), 968–971. 10.1126/science.277.5328.968 9252330

[phc312822-bib-0104] Rink, E. , & Wullimann, M. F. (2004). Connections of the ventral telencephalon (subpallium) in the zebrafish (Danio rerio). Brain Research, 1011(2), 206–220. 10.1016/j.brainres.2004.03.027 15157807

[phc312822-bib-0105] Rose, J. D. (2002). The neurobehavioral nature of fishes and the question of awareness and pain. Reviews in Fisheries Science, 10, 1–38. 10.1080/20026491051668

[phc312822-bib-0106] Rose, J. D. , Arlinghaus, R. , Cooke, S. J. , Diggles, B. K. , Sawynok, W. , Stevens, E. D. , & Wynne, C. D. (2014). Can fish really feel pain? Fish and Fisheries, 15(1), 97–133. 10.1111/faf.12010

[phc312822-bib-0107] Sandeman, D. C. , Kenning, M. , & Harzsch, S. (2014). Adaptive trends in malacostracan brain form and function related to behavior. In C. Derby & M. Thiel (Eds.), Crustacean nervous system and their control of behaviour (pp. 11–48). Oxford University Press.

[phc312822-bib-0108] Schwitzgebel, E. (2016). Phenomenal consciousness, defined and defended as innocently as I can manage. Journal of Consciousness Studies, 23, 224–235.

[phc312822-bib-0109] Shea, N. (2012). Methodological encounters with the phenomenal kind. Philosophy and Phenomenological Research, 84(2), 307–344. 10.1111/j.1933-1592.2010.00483.x 22654148 PMC3361722

[phc312822-bib-0110] Shea, N. , & Bayne, T. (2010). The vegetative state and the science of consciousness. The British Journal for the Philosophy of Science, 61(3), 459–484. 10.1093/bjps/axp046 22654125 PMC3361721

[phc312822-bib-0111] Shriver, A. (2018). The unpleasantness of pain for humans and other animals. In D. Bain , M. Brady , & J. Corns (Eds.), Philosophy of pain (pp. 147–162). Routledge.

[phc312822-bib-0112] Simon, J. (2020). Review of Carruthers, Human and animal minds: The consciousness questions laid to rest. In Notre dame philosophical reviews. Retrieved from https://ndpr.nd.edu/reviews/human‐and‐animal‐minds‐the‐consciousness‐questions‐laid‐to‐rest/

[phc312822-bib-0113] Singer, P. (1979). Practical ethics. Cambridge University Press. 39946524

[phc312822-bib-0114] Skora, L. I. , Yeomans, M. R. , Crombag, H. S. , & Scott, R. B. (2021). Evidence that instrumental conditioning requires conscious awareness in humans. Cognition, 208, 104546. 10.1016/j.cognition.2020.104546 33360281

[phc312822-bib-0115] Sneddon, L. U. (2003). The evidence for pain in fish: The use of morphine as an analgesic. Applied Animal Behaviour Science, 83(2), 153–162. 10.1016/S0168-1591(03)00113-8

[phc312822-bib-0116] Sneddon, L. U. (2004). Evolution of nociception in vertebrates: comparative analysis of lower vertebrates. Brain Research Reviews, 46(2), 123–130. 10.1016/j.brainresrev.2004.07.007 15464201

[phc312822-bib-0117] Sneddon, L. U. , Braithwaite, V. A. , & Gentle, M. J. (2003a). Do fishes have nociceptors? Evidence for the evolution of a vertebrate sensory system. Proceedings of the Royal Society of London Series B: Biological Sciences, 270(1520), 1115–1121. 10.1098/rspb.2003.2349 PMC1691351 12816648

[phc312822-bib-0118] Sneddon, L. U. , Braithwaite, V. A. , & Gentle, M. J. (2003b). Novel object test: Examining nociception and fear in the rainbow trout. The Journal of Pain, 4(8), 431–440. 10.1067/S1526-5900(03)00717-X 14622663

[phc312822-bib-0119] Sneddon, L. U. , Elwood, R. W. , Adamo, S. A. , & Leach, M. C. (2014). Defining and assessing animal pain. Animal Behaviour, 97, 201–212. 10.1016/j.anbehav.2014.09.007

[phc312822-bib-0120] Steel, D. (2015). Philosophy and the precautionary principle: science, evidence, and environmental policy. Cambridge University Press.

[phc312822-bib-0121] Sytsma, J . (2014). Attributions of consciousness. WIREs Cognitive Science, 5(6), 635–648. 10.1002/wcs.1320 26308870

[phc312822-bib-0122] Sytsma, J. , & Machery, E. (2010). Two conceptions of subjective experience. Philosophical Studies, 151(2), 299–327. 10.1007/s11098-009-9439-x

[phc312822-bib-0123] Sytsma, J. , & Machery, E. (2012). The Two Sources of Moral Standing. Review of Philosophy and Psychology 3(3), 303–324. 10.1007/s13164-012-0102-7

[phc312822-bib-0124] Szyszka, P. , Demmler, C. , Oemisch, M. , Sommer, L. , Biergans, S. , Birnbach, B. , Silbering, A. F. , & Galizia, C. G. (2011). Mind the gap: olfactory trace conditioning in honeybees. Journal of Neuroscience, 31(20), 7229–7239. 10.1523/jneurosci.6668-10.2011 21593307 PMC6622586

[phc312822-bib-0125] Travers, E. , Frith, C. D. , & Shea, N. (2018). Learning rapidly about the relevance of visual cues requires conscious awareness. Quarterly Journal of Experimental Psychology, 71(8), 1698–1713. 10.1080/17470218.2017.1373834 30027836

[phc312822-bib-0126] Tsuchiya, N. , Wilke, M. , Frässle, S. , & Lamme, V. A. (2015). No‐report paradigms: extracting the true neural correlates of consciousness. Trends in Cognitive Sciences, 19(12), 757–770. 10.1016/j.tics.2015.10.002 26585549

[phc312822-bib-0127] Tye, M. (2017). Tense bees and shell‐shocked crabs: Are animals conscious? Oxford University Press.

[phc312822-bib-0128] Varner, G. E. (2012). Personhood, ethics, and animal cognition: Situating animals in Hare's two level utilitarianism. Oxford University Press.

[phc312822-bib-0129] Watkins, S. , & Rees, G. (2007). The human superior colliculus: Neither necessary, nor sufficient for consciousness? Behavioral and Brain Sciences, 30(1), 108. 10.1017/S0140525X0700115X

[phc312822-bib-0130] Woodruff, M. L. (2017). Scientific uncertainty and the animal sentience precautionary principle. Animal Sentience, 16, 11. 10.51291/2377-7478.1237

[phc312822-bib-0131] Zabala, N. A. , & Gómez, M. A. (1991). Morphine analgesia, tolerance and addiction in the cricket Pteronemobius sp.(Orthoptera, Insecta). Pharmacology Biochemistry and Behavior, 40(4), 887–891. 10.1016/0091-3057(91)90102-8 1816576

[phc312822-bib-0132] Zabala, N. A. , Miralto, A. , Maldonado, H. , Nunez, J. A. , Jaffe, K. , Calderon, L. D. C. , Sytsma, J. , & Machery, E. (1984). Opiate receptor in praying mantis: effect of morphine and naloxone. Pharmacology Biochemistry and Behavior, 20, 683–687. 10.1007/s13164-012-0102-7 6330763

